# Dosimetric impact of different CT datasets for stereotactic treatment planning using 3D conformal radiotherapy or volumetric modulated arc therapy

**DOI:** 10.1186/s13014-015-0557-7

**Published:** 2015-12-01

**Authors:** Markus Oechsner, Leonhard Odersky, Johannes Berndt, Stephanie Elisabeth Combs, Jan Jakob Wilkens, Marciana Nona Duma

**Affiliations:** Department of Radiation Oncology, Klinikum rechts der Isar, Technische Universität München, Ismaninger Str. 22, 81675 Munich, Germany; Department of Electrical Engineering and Information Technology, Technische Universität München, Munich, Germany; Strahlentherapie Freising, Freising, Germany; Institute of Innovative Radiotherapy (iRT), Helmholtz Zentrum München, Munich, Germany

**Keywords:** SBRT, Average intensity projection, Maximum intensity projection, Mid-ventilation, VMAT

## Abstract

**Background:**

The purpose of this study was to assess the impact on dose to the planning target volume (PTV) and organs at risk (OAR) by using four differently generated CT datasets for dose calculation in stereotactic body radiotherapy (SBRT) of lung and liver tumors. Additionally, dose differences between 3D conformal radiotherapy and volumetric modulated arc therapy (VMAT) plans calculated on these CT datasets were determined.

**Methods:**

Twenty SBRT patients, ten lung cases and ten liver cases, were retrospectively selected for this study. Treatment plans were optimized on average intensity projection (AIP) CTs using 3D conformal radiotherapy (3D-CRT) and volumetric modulated arc therapy (VMAT). Afterwards, the plans were copied to the planning CTs (PCT), maximum intensity projection (MIP) and mid-ventilation (MidV) CT datasets and dose was recalculated keeping all beam parameters and monitor units unchanged. Ipsilateral lung and liver volumes and dosimetric parameters for PTV (D_mean_, D_2_, D_98_, D_95_), ipsilateral lung and liver (D_mean_, V_30_, V_20_, V_10_) were determined and statistically analysed using Wilcoxon test.

**Results:**

Significant but small mean differences were found for PTV dose between the CTs (lung SBRT: ≤2.5 %; liver SBRT: ≤1.6 %). MIPs achieved the smallest lung and the largest liver volumes. OAR mean doses in MIP plans were distinctly smaller than in the other CT datasets. Furthermore, overlapping of tumors with the diaphragm results in underestimated ipsilateral lung dose in MIP plans. Best agreement was found between AIP and MidV (lung SBRT). Overall, differences in liver SBRT were smaller than in lung SBRT and VMAT plans achieved slightly smaller differences than 3D-CRT plans.

**Conclusions:**

Only small differences were found for PTV parameters between the four CT datasets. Larger differences occurred for the doses to organs at risk (ipsilateral lung, liver) especially for MIP plans. No relevant differences were observed between 3D-CRT or VMAT plans. MIP CTs are not appropriate for OAR dose assessment. PCT, AIP and MidV resulted in similar doses. If a 4DCT is acquired PCT can be omitted using AIP or MidV for treatment planning.

## Introduction

Stereotactic body radiotherapy (SBRT) is well established in the treatment of tumors and metastases of lung and liver [1–4]. Typically, patients are treated in a few sessions using high doses per fraction. To minimize dose to organs at risk (OAR), small safety margins for the PTV are applied, which demands high precision in localization of the tumor, patient positioning and delivery of radiotherapy. A slow 3D computer tomography (CT) scan during free respiration can be acquired as planning CT (PCT) for treatment planning [5, 6]. Movement of the tumor can be assessed by four-dimensional computer tomography (4DCT). Using this motion information different approaches have been proposed to generate the planning target volume (PTV). For the internal target volume (ITV) concept the outline of the tumor is merged over all breathing phases of the 4DCT and a margin is added to generate the PTV [7, 8]. Maximum intensity projection (MIP) or average intensity projection (AIP) CT datasets can be calculated from the 4DCT to delineate the tumor herein [9–11]. Another group suggested the generation of a mid-ventilation CT dataset (MidV) for target definition and treatment planning [12].

Mostly SBRT planning is performed on one of the previously mentioned CT datasets. Novel approaches use 4D planning on 4DCT datasets [13]. Deformable image registration tools are applied to map the dose on PCT or end exhalation phase [14, 15]. But 4D planning is time consuming and tools for deformable image registration are not widely spread. The dosimetric differences between 4D and 3D treatment planning seem to be small and may not be clinically relevant [16]. Therefore 3D planning on a single CT dataset is still common practice.

Up to now only a limited number of studies compared the differences between CT datasets for treatment planning [17, 18]. These studies reported only comparisons between CT datasets for lung SBRT using 3D conformal radiotherapy (3D-CRT).

Volumetric modulated arc therapy (VMAT) [19] is nowadays widely used and increasingly for SBRT due to its improved treatment efficiency [20–22].

The aim of this study was to compare the dosimetric impact of using four different CT datasets (PCT, MIP, AIP and MidV) for SBRT dose calculation. We compared the dosimetric impact for lung and liver SBRT concerning the dose to PTV and OARs (ipsilateral lung and liver). Additionally, the influence of two different planning techniques (3D-CRT and VMAT) on calculated dose was investigated.

## Material and methods

### Patients and CT datasets

Twenty SBRT patients from our clinic, ten lung cases and ten liver cases, were retrospectively selected for this study. Each patient received a slow 3D-CT for treatment planning (PCT). Additionally, a 4DCT scan was acquired to receive motion information for delineation of the planning target volume. Data acquisition was performed using a 16 Multi-slice Somatom Emotion scanner (Siemens medical solutions, Erlangen, Germany). Patient’s breathing curve was measured with the Real-Time Position Management system (Varian Medical Systems, Palo Alto, CA, USA) during 4DCT scanning. Afterwards, the breathing curves were transfered to the syngo software (Siemens medical solutions, Erlangen, Germany) which sorted the acquired data retrospectively due to the temporal correlation with the breathing curve. The data were sorted into 10 phase-bins resulting in 3D-datasets which depict the imaged volume as 10 phases over the respiratory cycle.

From the 10 phases of the 4DCT an average intensity projection (AIP) CT was calculated representing the mean intensity of each voxel over all phases. A maximum intensity projection (MIP) CT dataset was also calculated which shows the maximum intensity of each voxel over all phases. AIP and MIP CT datasets were calculated using self-written programs in Matlab (MathWorks, Natick, MA, USA).

According to the concept of mid-ventilation CT scan [12] the tumor motion was determined by delineating the tumor in all phases of the 4DCT and evaluating the center of mass coordinates. The time-weighted mean tumor position was determined and the phase of the 10 phases was selected as mid-ventilation CT (MidV) which was closest to this mean tumor position. Due to the low contrast between tumor and liver tissue it was not possible to delineate the tumor in all 4DCT phases for liver SBRT. Therefore the position of the diaphragm was determined for all phases and the phase of the 4DCT scan with the diaphragm position closest to the mean position was selected as MidV CT. It is well known that the whole liver does not move in a linear fashion [23]. Therefore this approach is only an estimation of the real mean tumor position.

The image resolution of the CT datasets created from the 4DCT (AIP, MIP, MidV) was 1.0x1.0x2.1 mm^3^. The PCT datasets had a resolution of 1.0x1.0x3.0 mm^3^.

### Contouring and treatment planning

Contouring and treatment planning was performed with the Eclipse 10 planning system (Varian Medical Systems, Palo Alto, CA, USA). The ITV concept was used in this study as it is the applied concept for SBRT treatments in our clinic. Gross tumor volumes (GTVs) were contoured in all phases of the 4DCT and an ITV was generated. PTVs were created by adding an uniform margin of 5 mm to the ITV. The mean volume of the PTVs over all patients for lung SBRT was 115.2 cm^3^ (range: 27.1 cm^3^ to 218.8 cm^3^) and 153.5 cm^3^ for liver SBRT (range: 44.4 cm^3^ to 202.3 cm^3^). Afterwards, the PTVs were copied to the four CT datasets. For lung SBRT, the total ipsilateral lung volume was contoured as OAR on all four CT datasets and the total liver volume for the liver SBRT cases.

Two treatment plans were optimized on the AIP CT dataset for each patient using 3D-CRT (7–9 coplanar fields) and VMAT (2–3 partial arcs) technique. The prescription dose was 7 Gy delivered in 5 fractions corresponding to the 60 % isodose level surrounding the PTV. 3D-CRT plans were normalized to a dose of 100 % at the beam isocenter. For VMAT plans the dose was normalized that the 60 % isodose covers 100 % of the PTV. All optimized plans had a dose maximum between 101 and 105 % in the center of the PTV. Dose calculation was performed using the AAA algorithm (version 10.0.28) and heterogeneity correction. All plans were calculated for treatment on a Clinac Trilogy linear accelerator equipped with a 120 HD MLC (Varian Medical Systems, Palo Alto, CA, USA). Each leaf bank has 60 leafs: the inner 30 have a leaf width of 2.5 mm; the outer 30 have a leaf width of 5.0 mm. A photon energy of 15 MV was used for all plans. The optimized plans were copied to the PCT, MIP and MidV CT datasets and dose was recalculated keeping all beam parameters and monitor units unchanged. This enables the evaluation of dosimetric differences between the four CT datasets which arise from different density values.

### Data evaluation

Dosimetric parameters for PTV (D_mean_, D_2_, D_98_, D_95_) and the ipsilateral lung and liver (D_mean_, V_30_, V_20_, V_10_) were determined on all CT datasets. Further, we assessed the absolute volume of the lung and the liver (V_lung_, V_liver_). All parameters were compared and statistically analyzed using Wilcoxon test. A *p*-value <0.05 was considered as statistically significant. The statistical analyses were performed using SPSS Software for Windows version 23.0 (SPSS Inc., Chicago, IL, USA).

## Results

### Dose to PTV

**Fig. 1:**
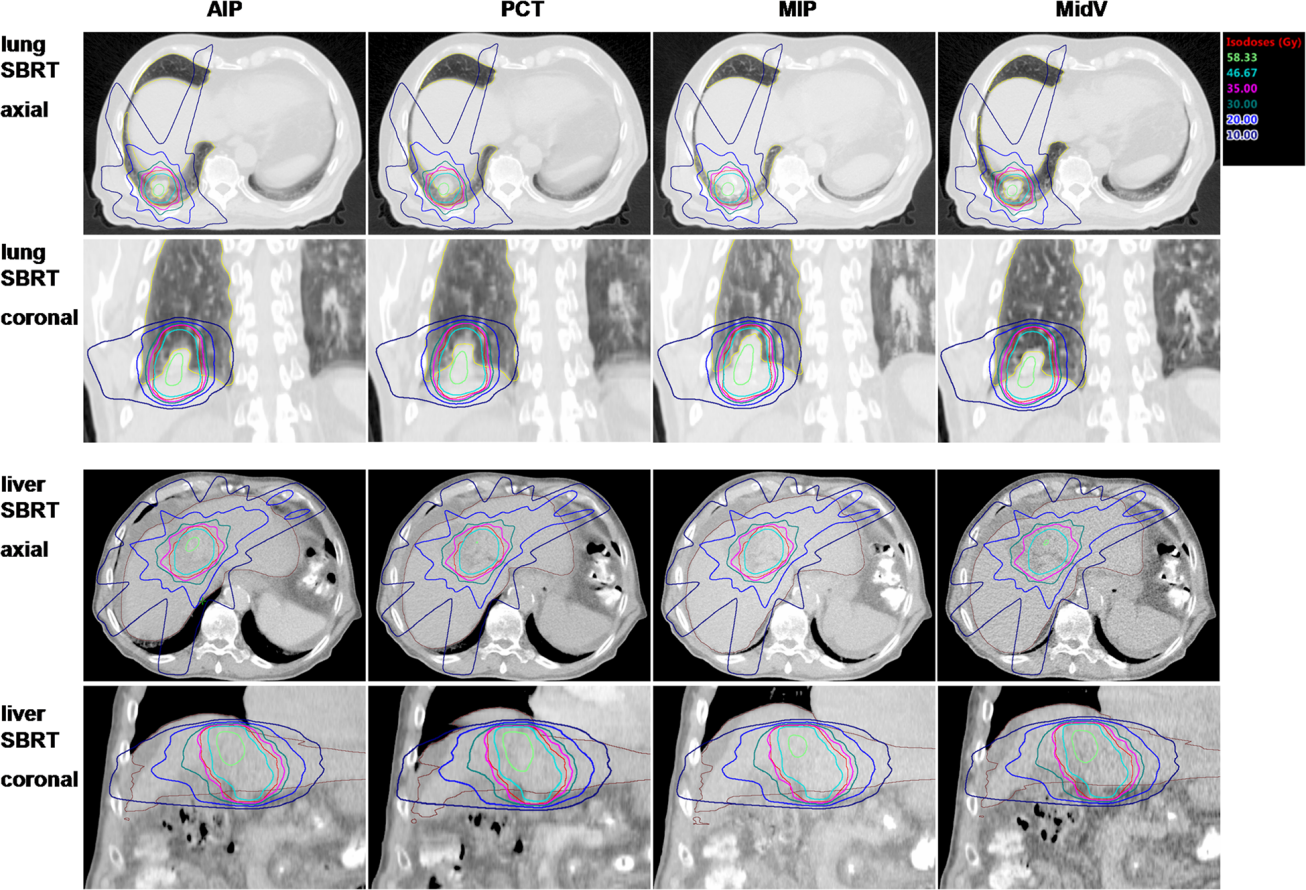
Axial and coronal slices with dose distributions for a lung and a liver SBRT patient. The size of lung and liver appears differently in the CT datasets. PCT: planning CT, AIP: average intensity projection CT, MIP: maximum intensity projection CT, MidV: mid-ventilation CT

In Fig. 1 axial slices of the four CT datasets with dose distributions for a lung and a liver SBRT patient are depicted. Relative differences of doses over all patients for the PTV are depicted in Table 1 (lung SBRT) and Table 2 (liver SBRT).

**Table 1:** PTV dose differences in lung SBRT between the CT datasets

		3D-CRT		VMAT	
	CT datasets	Δ [%]	*P* value	Δ [%]	*P* value
ΔD_mean_	PCT vs AIP	−0.8 ± 0.5	0.01^*^	−0.5 ± 0.5	0.01^*^
	MIP vs AIP	1.5 ± 1.4	0.01^*^	1.6 ± 1.5	0.01^*^
	MidV vs AIP	0.0 ± 0.3	0.96	−0.1 ± 0.3	0.72
	MIP vs PCT	2.4 ± 0.7	0.01^*^	2.2 ± 1.5	0.01^*^
	MidV vs PCT	0.8 ± 0.7	0.02^*^	0.5 ± 0.6	0.06
	MIP vs MidV	1.6 ± 1.3	0.01^*^	1.7 ± 1.5	0.01^*^
ΔD_2_	PCT vs AIP	−0.5 ± 0.7	0.04^*^	−0.2 ± 0.8	0.29
	MIP vs AIP	1.0 ± 1.5	0.07	1.6 ± 2.0	0.01^*^
	MidV vs AIP	−0.1 ± 0.4	0.39	0.0 ± 0.5	0.96
	MIP vs PCT	1.5 ± 2.0	0.01^*^	1.9 ± 1.9	0.01^*^
	MidV vs PCT	0.4 ± 0.9	0.13	0.2 ± 1.1	0.33
	MIP vs MidV	1.1 ± 1.6	0.06	1.7 ± 2.0	0.01^*^
ΔD_98_	PCT vs AIP	−1.4 ± 0.4	0.01^*^	−1.1 ± 0.7	0.01^*^
	MIP vs AIP	1.0 ± 1.6	0.07	1.0 ± 1.4	0.02^*^
	MidV vs AIP	0.2 ± 0.4	0.09	0.0 ± 0.4	0.59
	MIP vs PCT	2.4 ± 1.9	0.01^*^	2.2 ± 1.7	0.01^*^
	MidV vs PCT	1.7 ± 0.6	0.01^*^	1.1 ± 0.7	0.01^*^
	MIP vs MidV	0.7 ± 1.5	0.20	1.0 ± 1.4	0.07
ΔD_95_	PCT vs AIP	−1.3 ± 0.3	0.01^*^	−0.9 ± 0.4	0.01^*^
	MIP vs AIP	1.2 ± 1.6	0.02^*^	1.0 ± 1.4	0.03^*^
	MidV vs AIP	0.2 ± 0.4	0.09	0.0 ± 0.4	0.68
	MIP vs PCT	2.5 ± 1.8	0.01^*^	1.9 ± 1.6	0.01^*^
	MidV vs PCT	1.5 ± 0.5	0.01^*^	0.9 ± 0.4	0.01^*^
	MIP vs MidV	1.0 ± 1.4	0.04^*^	1.0 ± 1.5	0.06

Relative differences over all patients (mean ± standard deviation) of dosimetric values for PTV in lung SBRT between the different CT datasets for 3D-CRT and VMAT plans. *PCT* planning CT, *AIP* average intensity projection CT, *MIP* maximum intensity projection CT, *MidV* mid-ventilation CT. *assigns statistical significant values (*p* < 0.05)

**Table 2:** PTV dose differences in liver SBRT between the CT datasets

		3D-CRT		VMAT	
	CT datasets	Δ [%]	*P* value	Δ [%]	*P* value
ΔD_mean_	PCT vs AIP	−0.5 ± 0.7	0.04^*^	−0.4 ± 0.5	0.03^*^
	MIP vs AIP	−1.1 ± 0.7	0.01^*^	−0.9 ± 0.7	0.01^*^
	MidV vs AIP	0.0 ± 0.3	0.39	0.1 ± 0.4	0.24
	MIP vs PCT	−0.7 ± 0.9	0.06	−0.5 ± 0.7	0.07
	MidV vs PCT	0.4 ± 0.8	0.06	0.5 ± 0.6	0.05
	MIP vs MidV	−1.1 ± 0.7	0.01^*^	−0.9 ± 0.5	0.01^*^
ΔD_2_	PCT vs AIP	−0.3 ± 0.4	0.05	−0.3 ± 0.5	0.06
	MIP vs AIP	−1.2 ± 0.7	0.01^*^	−1.1 ± 0.6	0.01^*^
	MidV vs AIP	−0.2 ± 0.4	0.14	−0.1 ± 0.4	0.67
	MIP vs PCT	−0.8 ± 0.8	0.01^*^	−0.7 ± 0.7	0.01^*^
	MidV vs PCT	0.1 ± 0.7	0.48	0.2 ± 0.7	0.33
	MIP vs MidV	−0.9 ± 0.6	0.01^*^	−0.9 ± 0.5	0.01^*^
ΔD_98_	PCT vs AIP	−0.8 ± 0.9	0.02^*^	−0.5 ± 0.4	0.01^*^
	MIP vs AIP	−0.6 ± 1.3	0.06	−0.8 ± 0.6	0.01^*^
	MidV vs AIP	0.4 ± 0.5	0.03^*^	0.1 ± 0.3	0.15
	MIP vs PCT	0.2 ± 0.9	0.92	−0.4 ± 0.7	0.10
	MidV vs PCT	1.2 ± 1.0	0.01^*^	0.6 ± 0.5	0.02^*^
	MIP vs MidV	−1.0 ± 1.2	0.01^*^	−0.9 ± 0.6	0.01^*^
ΔD_95_	PCT vs AIP	−0.7 ± 0.8	0.02^*^	−0.5 ± 0.4	0.02^*^
	MIP vs AIP	−1.2 ± 1.7	0.07	−0.8 ± 0.6	0.01^*^
	MidV vs AIP	0.4 ± 0.6	0.04^*^	0.1 ± 0.3	0.08
	MIP vs PCT	−0.5 ± 1.9	0.72	−0.4 ± 0.7	0.14
	MidV vs PCT	1.1 ± 0.9	0.01^*^	0.6 ± 0.6	0.02^*^
	MIP vs MidV	−1.6 ± 1.3	0.01^*^	−0.9 ± 0.5	0.01^*^

Relative differences over all patients (mean ± standard deviation) of dosimetric values for PTV in liver SBRT between the different CT datasets for 3D-CRT and VMAT plans. *PCT* planning CT, *AIP* average intensity projection CT, *MIP* maximum intensity projection CT, *MidV* mid-ventilation CT. *assigns statistical significant values (*p* < 0.05)

For the lung SBRT the largest mean difference was found for 3D-CRT plans and parameter D_95_ (MIP vs. PCT: 2.5 ± 1.8 %). The differences between CT datasets were ≤2.5 % (3D-CRT) or ≤2.2 % (VMAT). Nevertheless, many values showed significant differences (*p* < 0.05, Table 1). AIP and MidV achieved the best agreements over all parameters. Over all patients, the mean difference for all parameters was somewhat higher in MIP CTs compared to the others.

The liver SBRT plans showed smaller dose differences for the PTV between the CT datasets than the lung SBRT plans (Table 2). The largest differences were found for 3D-CRT plans and D_95_ (MIP vs. MidV: −1.6 ± 1.3 %). All other differences between CT datasets were ≤1.2 %. AIP and MidV CT resulted in the best agreements over all parameters. In comparison to 3D-CRT plans VMAT showed slightly smaller differences over all parameters.

### Organ volume

**Fig. 2:**
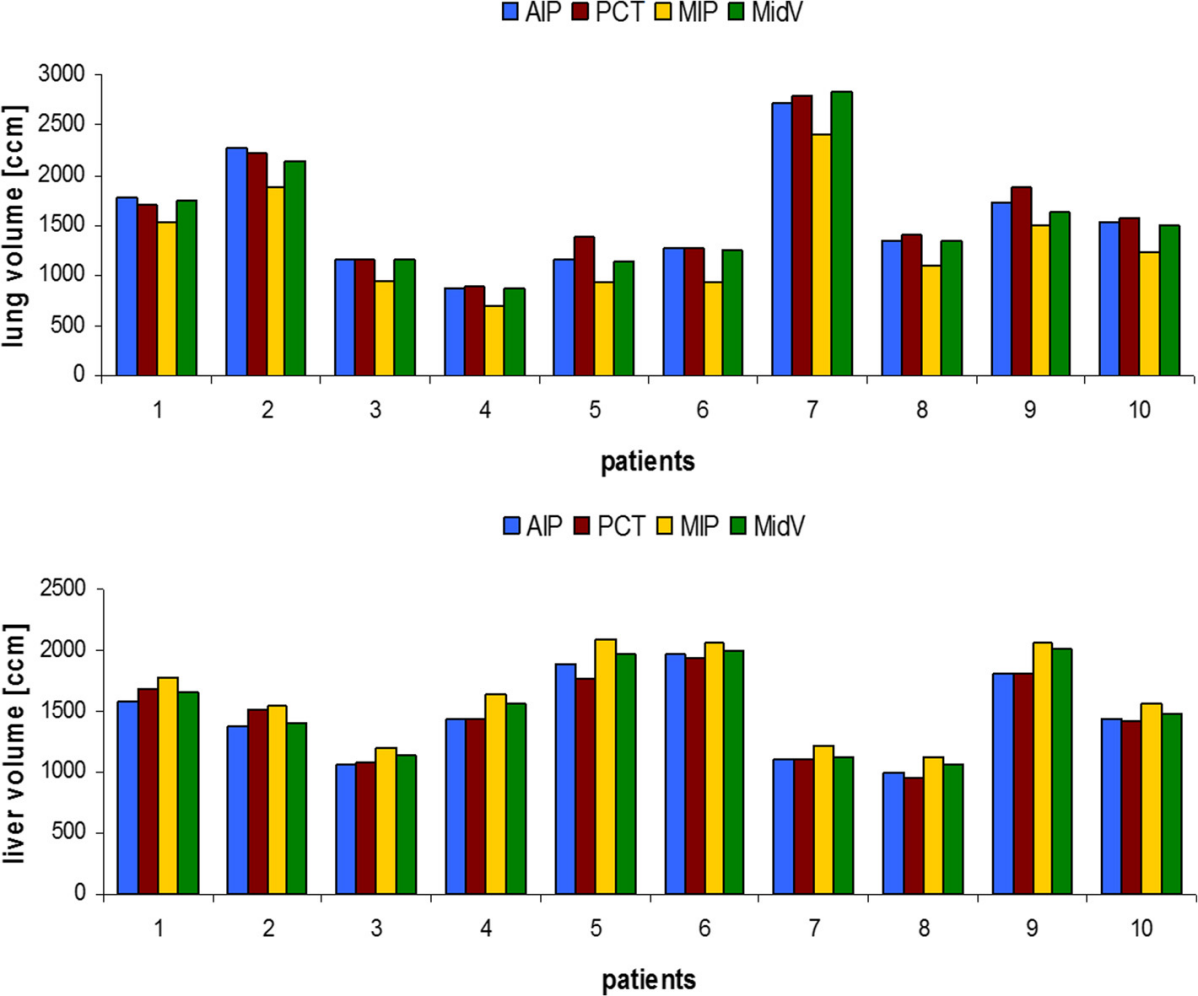
Lung and a liver volumes of all patients. In MIP CTs the determined lung volume was always smaller and the liver volume was always larger than in the other CT datasets. AIP: average intensity projection CT, PCT: planning CT, MIP: maximum intensity projection CT, MidV: mid-ventilation CT

Figure 2 shows the lung and liver volumes of all patients. In MIP CTs the lung volume was significantly smaller than in all other CT datasets (*p* ≤ 0.01). The largest volumes were contoured in PCTs but that was not statistically significant. The best agreement for lung volumes was found between MidV and AIP CTs (−1.5 ± 3.0 %). For the liver SBRT cases, the largest liver volume was always contoured in MIP CTs (*p* ≤ 0.01). The smallest differences were found between PCT and AIP (0.5 ± 4.8 %). PCT and AIP achieved also the smallest liver volumes as compared to the other CT datasets.

### Dose to organs at risk

**Fig. 3:**
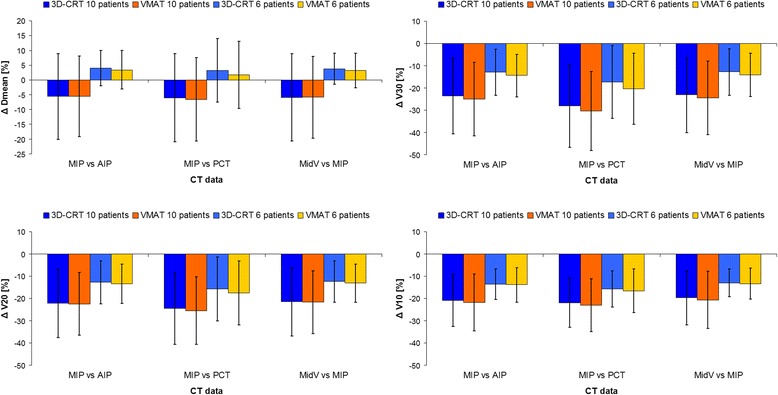
Analysis of lung dose by tumor location. Bars show the mean difference (±standard deviation) in lung D_mean_ and V_x_ over all patients between MIP and the other CT datasets. Mean values over all 10 patients resulted in large differences. If patients with tumor overlap to the diaphragm were excluded (6 patients left), the differences decreased drastically. AIP: average intensity projection CT, PCT: planning CT, MIP: maximum intensity projection CT, MidV: mid-ventilation CT

Relative differences for the ipsilateral lung dose over all patients are listed in Table 3. For the difference of absolute lung volumes receiving a certain dose (V_x_), best agreement was found between AIP and MidV with mean differences ≤1.2 %. PCT achieved up to 8.9 % differences (VMAT) to AIP and MidV with smaller differences with decreasing dose. In MIP plans the absolute volumes receiving certain doses are significantly smaller than in all other CT datasets. Only small differences (≤2.4 %) were found for lung parameters between 3D-CRT and VMAT plans. No significant differences were found for lung D_mean_ between AIP, PCT and MidV. MIP plans resulted in distinctly lower lung D_mean_. As the results were unexpected (smaller lung volumes in MIP resulting in smaller lung D_mean_ in MIP), we analyzed the CTs by tumor location. In 4 out of 10 patients the PTV partially overlaps with the diaphragm. If these patients were excluded from the analysis (Fig. 3) lung D_mean_ in MIP plans was higher than in plans on the other CT datasets (3.3–4.0 %). The difference in lung volume receiving a certain dose (V_x_) between MIP and the other CT datasets decreased distinctly.

**Table 3:** Differences for ipsilateral lung dose in lung SBRT

		3D-CRT		VMAT	
	CT datasets	Δ [%]	*P* value	Δ [%]	*P* value
ΔD_mean_	PCT vs AIP	0.6 ± 6.6	0.39	1.2 ± 6.8	0.29
	MIP vs AIP	−5.6 ± 14.5	0.21	−5.6 ± 13.7	0.20
	MidV vs AIP	0.4 ± 2.8	0.80	0.4 ± 2.7	0.80
	MIP vs PCT	−6.0 ± 14.9	0.13	−6.5 ± 14.1	0.06
	MidV vs PCT	0.2 ± 7.7	0.39	−0.4 ± 7.7	0.24
	MIP vs MidV	−5.9 ± 14.7	0.28	−5.8 ± 13.9	0.14
ΔV_30_	PCT vs AIP	7.2 ± 12.7	0.07	8.9 ± 13.5	0.07
	MIP vs AIP	−23.6 ± 17.0	0.01^*^	−25.0 ± 16.5	0.01^*^
	MidV vs AIP	−0.6 ± 2.3	0.39	−0.6 ± 2.5	0.33
	MIP vs PCT	−28.0 ± 18.5	0.01^*^	−30.4 ± 17.7	0.01^*^
	MidV vs PCT	−6.1 ± 11.2	0.07	−7.4 ± 11.6	0.06
	MIP vs MidV	−23.1 ± 16.9	0.01^*^	−24.5 ± 16.5	0.01^*^
ΔV_20_	PCT vs AIP	3.5 ± 10.2	0.07	4.9 ± 10.6	0.07
	MIP vs AIP	−22.2 ± 15.4	0.01^*^	−22.4 ± 14.1	0.01^*^
	MidV vs AIP	−0.9 ± 3.4	0.17	−0.9 ± 3.4	0.17
	MIP vs PCT	−24.4 ± 16.1	0.01^*^	−25.5 ± 15.2	0.01^*^
	MidV vs PCT	−3.3 ± 10.7	0.07	−4.6 ± 10.6	0.07
	MIP vs MidV	−21.4 ± 15.4	0.01^*^	−21.6 ± 14.1	0.01^*^
ΔV_10_	PCT vs AIP	1.6 ± 9.1	0.24	1.9 ± 9.8	0.29
	MIP vs AIP	−20.8 ± 11.7	0.01^*^	−21.8 ± 12.7	0.01^*^
	MidV vs AIP	−1.2 ± 4.3	0.17	−1.2 ± 4.5	0.20
	MIP vs PCT	−21.8 ± 11.1	0.01^*^	−23.0 ± 11.9	0.01^*^
	MidV vs PCT	−2.0 ± 10.2	0.11	−2.2 ± 10.9	0.14
	MIP vs MidV	−19.7 ± 12.1	0.01^*^	−20.7 ± 12.8	0.01^*^

Relative differences for ipsilateral lung dose (mean ± standard deviation) in lung SBRT between the different CT datasets for 3D-CRT and VMAT plans. *PCT* planning CT, *AIP* average intensity projection CT, *MIP* maximum intensity projection CT, *MidV* mid-ventilation CT. *assigns statistical significant values (*p* < 0.05)

In liver SBRT (Table 4), MIP plans achieved smaller liver D_mean_ values than plans calculated on the other CT datasets. The difference for V_30_, V_20_ and V_10_ was always ≤2.5 % between AIP, MidV and PCT. MIP plans resulted in somewhat larger differences for V_x_ (≤5.4 %). 3D-CRT and VMAT plans achieved nearly the same results for all CT datasets and OAR dose parameters (Δ ≤1.0 %).

**Table 4:** Differences for liver dose in liver SBRT

		3D-CRT		VMAT	
	CT datasets	Δ [%]	*P* value	Δ [%]	*P* value
ΔD_mean_	PCT vs AIP	0.9 ± 3.8	0.88	0.9 ± 3.9	0.76
	MIP vs AIP	−5.8 ± 4.7	0.04^*^	−5.3 ± 4.6	0.05
	MidV vs AIP	−2.3 ± 3.4	0.07	−2.1 ± 3.3	0.08
	MIP vs PCT	−6.6 ± 3.0	0.01^*^	−6.2 ± 2.9	0.01^*^
	MidV vs PCT	−3.1 ± 3.2	0.03^*^	−3.0 ± 3.3	0.04^*^
	MIP vs MidV	−3.6 ± 2.7	0.01^*^	−3.2 ± 2.7	0.01^*^
ΔV_30_	PCT vs AIP	1.7 ± 4.9	0.88	1.5 ± 4.7	0.72
	MIP vs AIP	3.6 ± 8.5	0.88	3.9 ± 8.4	0.96
	MidV vs AIP	2.5 ± 5.1	0.20	2.3 ± 5.2	0.58
	MIP vs PCT	1.8 ± 5.6	0.80	2.3 ± 5.5	0.58
	MidV vs PCT	0.9 ± 3.5	0.45	0.8 ± 3.3	0.58
	MIP vs MidV	0.9 ± 3.7	0.72	1.4 ± 3.4	0.80
ΔV_20_	PCT vs AIP	1.6 ± 5.5	0.80	1.0 ± 5.2	0.45
	MIP vs AIP	4.1 ± 7.7	0.14	4.4 ± 7.5	0.09
	MidV vs AIP	2.4 ± 4.1	0.17	2.4 ± 4.6	0.24
	MIP vs PCT	2.4 ± 4.4	0.17	3.4 ± 5.9	0.05
	MidV vs PCT	0.8 ± 3.3	0.33	1.5 ± 4.2	0.39
	MIP vs MidV	1.6 ± 3.7	0.51	1.9 ± 3.2	0.33
ΔV_10_	PCT vs AIP	1.3 ± 4.7	0.96	1.1 ± 4.9	0.65
	MIP vs AIP	5.2 ± 5.3	0.01^*^	5.4 ± 5.6	0.01^*^
	MidV vs AIP	2.4 ± 3.1	0.02^*^	2.5 ± 3.3	0.04^*^
	MIP vs PCT	4.0 ± 4.5	0.03^*^	4.3 ± 4.3	0.03^*^
	MidV vs PCT	1.3 ± 4.2	0.45	1.5 ± 4.2	0.33
	MIP vs MidV	2.7 ± 2.8	0.03^*^	2.8 ± 3.1	0.04^*^

Relative differences for liver dose over all patients (mean ± standard deviation) in liver SBRT between the different CT datasets for 3D-CRT and VMAT plans. *PCT* planning CT, *AIP* average intensity projection CT, *MIP* maximum intensity projection CT, *MidV* mid-ventilation CT. *assigns statistical significant values (*p* < 0.05)

## Discussion

For stereotactic treatment of lung and liver tumors several CT datasets are acquired (PCT, 4DCT) or can be generated (MIP, AIP, MidV) for treatment planning. In this study the impact of four different CT datasets for treatment planning on dose to PTV and OARs was evaluated for lung and liver SBRT cases using 3D-CRT or VMAT.

PTV doses in lung SBRT showed only small but partially statistically significant differences between the treatment plans on different CT datasets regardless of the chosen technique (3D-CRT or VMAT), with the largest mean difference of 2.5 % (D_95_). Similar data are available in literature for 3D-CRT SBRT. Tian et al. [18] compared dosimetric differences in lung SBRT between 3D-CRT plans calculated on AIP, MIP and PCT datasets. They reported small but significant dose differences for PTV between the CT datasets. They mentioned that these small differences may not reflect clinical significant changes.

For the liver SBRT cases dose differences for PTV between the CT datasets were even smaller (≤1.6 %) than for lung SBRT. This might be due to the negligible density differences between soft tissues in the abdomen, which are furthermore also significantly smaller than in the thorax. Therefore different tissue representations in the CT datasets have less effect on calculated PTV doses in liver SBRT.

Nonetheless, if any of these four CT datasets is selected for treatment planning, a treatment plan will be calculated which fulfills the dose constraints for PTV dose coverage on either of the CT datasets. But, the CT dataset has a major impact on the contoured lung and liver volume and hence on dosimetric parameters for OARs. Therefore OAR dose as depicted by the CT datasets might be a major issue. MIP CTs always achieved the smallest lung volume and the largest liver volume. This is a consequence of the image generation of MIP by using the highest voxel intensities of the 4DCT phases. Differences between the other CT datasets for lung volumes were also found but these were small and not statistically significant. For the liver cases MidV CT achieved significantly larger liver volumes than PCT and AIP.

Concerning the lung volume receiving a certain dose (V_x_), MIP plans achieved distinctly smaller values than all other plans. Except for V_30_ (PCT), the other CT datasets showed no significant differences in V_x_.

In this work and also in Ref [18] V_x_ values were determined as absolute organ volumes receiving a certain dose. In clinical practise V_x_ is often referred to relative organ volumes. This would result in smaller differences between V_x_ of MIP and the other CT datasets because of the smaller lung volumes in MIP images.

Another important clinical dose parameter for the lung is D_mean_, which was not reported in Ref [18]. Likewise, D_mean_ showed no significant differences between AIP, PCT and MidV, but larger differences for MIP (−5.6 to−6.5 %) over all patients. Han et al. [17] compared dose to the lung (lung-ITV) between PCT and AIP. They did not find significant differences in OAR dose (D_mean_ and D_max_) between PCT and AIP datasets. This is in accordance to our findings for PCT and AIP.

Furthermore, the impact of tumor location on dose differences between the CT datasets was analysed in our study. In MIP plans the tumor location can have a large impact on lung dose (Fig. 3). Over all patients lung D_mean_ in MIP plans was about 6 % smaller than in the other plans. If the four lung cases in which the tumor overlaps with the diaphragm were excluded from the analysis, MIP plans achieved on average higher lung D_mean_ values than plans of the other datasets. Differences for V_x_ are also distinctly reduced. This could be seen in 3D-CRT as well as in VMAT plans. This effect wasn’t found for AIP, PCT and MidV CT datasets. For the liver SBRT cases no such effect of tumor location could be seen in our data. This effect is obviously due to the image generation of MIP. Lung near the diaphragm is replaced by liver which results in lower lung dose. However, the number of patients in this study is too small for a detailed analysis of the influence of tumor location on dose differences between the CT datasets.

Distinctly less publication are available on liver SBRT. To our knowledge no study compared the influence of different CT datasets on dose in liver SBRT. Over all patients MIP plans resulted in the smallest mean liver dose. This is due to the largest contoured liver volume in MIP CTs. Otherwise MIP achieved larger Vx values than the other CT datasets, because of Vx are related to absolute OAR volumes in this work.

Compared to the lung, the liver D_mean_ between MidV and AIP or PCT showed larger differences (−2.3 to−3.1 %). This is due to the significant larger liver volume contoured in MidV CTs. Statistically significant differences for liver volumes receiving a certain dose in MidV CTs were only found for V_10_.

VMAT is nowadays widely used and increasingly for SBRT. Several studies reported application of VMAT for lung and/or liver SBRT [21, 22, 24, 25] noting good dose coverage, sparing of OARs and reduced dose delivery times. The influence of different CT datasets for dose calculation between 3D-CRT and VMAT plans showed only small differences for PTV dose parameters between both techniques (≤0.6 % in lung SBRT, ≤0.7 % in liver SBRT). Differences in OAR doses were also small (<2.4 %). Overall VMAT showed somewhat smaller dose differences compared to 3D-CRT. This could be due to the fact, that in VMAT the beam paths through the body are spread over a larger area and local differences in tissue densities between the CT datasets have less effect on dose distribution.

When 4DCT was introduced into the routine treatment planning and became widely available, the question that came up was whether we could trust the planning performed on the CT datasets reconstructed from the 4DCT. The simplest way to assess the differences in these CTs was to copy the plans and recalculate the dose. If 4DCT is available, the AIP is one of the options for treatment planning. All plans in this study were planned on the AIP CT and then copied to the other CT datasets. We intended to evaluate dose differences which arise only from density differences between the CT datasets. Due to the similar PTV dose coverage in all four CT datasets we expect that only very small changes are necessary to adapt the plans to PCT, MIP or MidV CTs. As already mentioned in the discussion the major issue is the dose to OARs. These are always depicted differently in the CT datasets resulting in different contoured volumes and thus in different calculated doses.

One limitation of our study was the dose calculation with an AAA algorithm. More sophisticated algorithms like Monte Carlo based methods or the Acuros XB from Varian [26, 27] achieve higher accuracy especially in areas with high gradients in electron density (i.e. lung). AAA tends to overestimate the dose in these regions. If lung SBRT plans are calculated with AAA and Acuros, the D_98_ [28] can differ up to +12.3 % for AAA whereas D_mean_ shows only small differences [28, 29]. However a recalculation with Acuros is expected to have only a small impact on the dose for liver SBRT.

Which CT dataset might be most suitable for treatment planning in lung or liver SBRT? Overall, differences in PTV dose parameters were small between the four CT datasets. The delineation of OAR volumes in the MIP dataset resulted in large differences with underestimation (lung) or overestimation (liver) of volumes due to the image generation properties. This again has an influence on the calculated OAR mean dose and V_x_ which differ significantly compared to the other CT datasets. Therefore, MIP is not suitable for treatment planning of lung and liver SBRT. Several work compared PTV volume definition by using different CT datasets [9–11]. Depending on the clinical practise in the department it might be helpful to have more than one CT dataset at hand for the whole therapy planning process.

The PCT achieved good agreement to AIP and MidV CT for PTV coverage. OAR volumes in the PCT are in accordance to AIP volumes (lung and liver) and MidV volumes (lung). Significant differences between PCT and MidV were found in mean dose to the liver. As already mentioned in Ref. [17, 18] image quality in free breathing PCT is often reduced due to motion. Furthermore, if a 4DCT scan is acquired anyway to receive motion information of the tumor, the acquisition of an additional PCT seems to be unnecessary. This would spare the patient from additional CT dose. Under such circumstances a PCT scan is not preferable to AIP or MidV.

The MidV plans achieved good agreement to AIP for PTV doses, lung volumes and lung doses. Significant differences were found for liver volume and liver V_10_. The MidV CT as used in this work requires determination of the time weighted mean tumor position in the 4DCT by contouring the GTVs in every phase. The mid-ventilation approach [12] uses the exact phase of the 4DCT which is closest to the mean tumor position. In our work we selected the one of 10 evenly distributed phases as mid-ventilation CT (MidV) with the tumor position closest to the time weighted mean tumor position. This is only a rough estimate but we would expect only negligible effects for dose calculation. A refinement of the MidV concept is the reconstruction of a mid-position CT (MidP) [30]. This concept was recently applied for liver SBRT [31]. The MidP requires deformable image registration methods and is beyond the scope of our work.

The AIP-CT achieved good agreement for PTV dose to MidV and PCT plans. For the lung cases AIP and MidV achieved nearly the same results. The AIP reconstruction from the 4DCT dataset is straight forward and available on a variety of commercial software. Assuming a good image quality of the 4DCT, using AIP for treatment planning needs less effort and creates reliable results for dose calculation of PTV and OARs.

For clinical routine one other issue to be taken into consideration is the daily setup correction by image guidance. In clinical practice the CT dataset used for treatment planning is usually the CT dataset used also for alignment with the linac on board imaging - e.g. cone beam CTs (CBCT). As reported in Ref. [32], using AIP or MIP CT datasets for alignment to CBCTs can result in different shifts especially for large moving tumors or asymmetrical breathing patterns.

## Conclusions

The dosimetric impact of using four different CT datasets for dose calculation showed only small differences for the dose to the PTV. No differences were observed between 3D-CRT or VMAT plans with respect to the chosen CT dataset. Larger differences were found for the dose to the organs at risk (ipsilateral lung, liver) especially for MIP plans. MIP plans seem not to be reliable for OAR dose assessment and should not be used for treatment planning. PCT, AIP and MidV resulted in similar doses and are all applicable. Assuming a 4DCT is acquired in any case, an additional PCT can be omitted using AIP or MidV for treatment planning.
